# The relationship between cyber upward social comparison and cyberbullying behaviors: A moderated mediating model

**DOI:** 10.3389/fpsyg.2022.1017775

**Published:** 2022-11-21

**Authors:** Hong Wen, Xiangwei Kong, Yonggang Feng

**Affiliations:** ^1^Faculty of Education, Shandong Normal University, Jinan, China; ^2^Department of Psychology, Central China Normal University, Wuhan, China

**Keywords:** cyber upward social comparison, cyberbullying, moral justification, online social support, moderated mediating model

## Abstract

Based on the General Strain Theory and the moderating role model of social support, the present study explored the relationship between cyber upward social comparison and cyberbullying and further explored the mediating role of moral justification and the moderating role of online social support. This model was examined with 660 Chinese college students. Participants completed questionnaires regarding cyber upward social comparison, cyberbullying, moral justification, and online social support. After basic demographic variables were controlled, cyber upward social comparison was significantly and positively associated with cyberbullying. Moral justification played a mediating role in the relationship between cyber upward social comparison and cyberbullying. The mediating effect of moral justification on the relationship between cyber upward social comparison and cyberbullying was moderated by online social support. The results of this study will provide references for the prevention and intervention of cyberbullying.

## Introduction

In recent years, the Internet has become an indispensable part of modern life. According to the 47th “Statistical Report on China’s Internet Network Development” issued by the China Internet Information Center, as of December 2020, the number of Chinese internet users has reached 989 million, the internet penetration rate has reached 70.4%, and the proportion of young people has reached 21.0% (China Internet Information Center, 2021). Social media networks bring not only many conveniences but also negative online behaviors that cannot be ignored, such as cyberbullying, which has a negative impact on individual development (Brandau and Evanson, 2018; Kumar and Goldstein, 2020). Cyberbullying is a deliberate and repeated attack by individuals or groups against victims through electronic communication (Sticca and Perren, 2013). Compared with traditional bullying behaviors, the online environment eliminates the time and space constraints of bullying, offensive videos/pictures and comments were published any time or anywhere, and remained on social networking platforms for a long time. Further, cyberbullying information could be spread quickly and persist over time (Berne et al., 2019), which could inflict significant harm to psychological health (e.g., distress, negative emotions, anxiety, reduced self-esteem, suicidal ideation, etc.; Aboujaoude et al., 2015). Relevant studies have shown that cyberbullying behaviors are more common and more harmful than traditional bullying behaviors (Lester et al., 2012; Eyuboglu et al., 2021). In many countries, about 11 to 42% of children and adolescents have experienced cyberbullying, and the incidence of cyberbullying among adolescents and children is increasing with the popularity of the Internet (Kraft, 2006; Aboujaoude et al., 2015). Additionally, they have a great impact on the mental health of bullied individuals (Juvonen and Gross, 2008; Fletcher et al., 2014; Zych et al., 2015). Therefore, analyzing the antecedent variables of cyberbullying and determining the underlying mechanism will make it more efficient to take active protective measures and help individuals avoid being cyberbullied and build a healthy and harmonious online life.

Social networking platform is characterized by a convenient, immediate, and cross-regional nature, which enables people to obtain access to a larger amount of information released by others than in the past. Social media is used to build online presences and social networks, with impression management, people might selectively express their real values and identities, which leads to the fact that other people would be influenced by individuals’ level of expressing “true self” (Tosun, 2012). People unconsciously make social comparisons when browsing information, and upward social comparisons often have a negative impact on people (Twenge, 2019). Studies have shown that compared with face-to-face comparisons, upward comparisons which refer to individuals comparing themselves with those who are better off (Festinger, 1954; Zheng et al., 2020) through social media will bring more negative emotions (Fardouly et al., 2017), which may lead to cyberbullying. Many previous studies have explored the factors influencing cyberbullying from the perspective of individuals themselves, such as self-esteem, compassion and prosociality, and found that there is a significant relationship between low self-esteem and cyberbullying (Aliyev and Gengec, 2019; Lei et al., 2020; Pascual-Sanchez et al., 2021), both empathy and prosociality are effective in reducing cyberbullying (Ferreira et al., 2021; Xu and Wang, 2021). Few studies have examined the effects of online social comparison on cyberbullying from an environmental perspective (Geng et al., 2021). This research is the first to explore the relationship between cyber upward social comparison and cyberbullying. Individuals who conduct upward social comparisons may generate moral justification, thereby further promoting cyberbullying behaviors. In addition, social support in individuals’ network environments has an important impact on the generation and development of cyberbullying behavior. When individuals receive online social support, they may better deal with the negative effects of cyber incidents and have an impact on cyberbullying behavior. Given that previous studies mainly focused on the influencing factors of cyberbullying and there are few studies on the impact on the environment (Bai et al., 2020; De Pasquale et al., 2021), this study was designed to explore the moderating effect of online social support. In summary, this research intends to use college students as the research participants and comprehensively investigate the prediction and mechanism of online social comparison, moral justification, and online social support on cyberbullying behavior in the context of Chinese culture to enrich relevant research in this field.

### Cyber upward social comparison and cyberbullying

Social comparison is a ubiquitous social psychological phenomenon, and individuals often evaluate and recognize themselves through social comparison (Wang D. et al., 2016). According to the different directions of comparison, social comparison can be divided into upward social comparison and downward social comparison. For example, upward social comparison occurs when a person compares them-self or them-self to a superior person; downward social comparison occurs when a person compares them-self or them-self to others who are inferior to him or her. Cyber social comparison is an extension of real-life social comparison in social networking platforms. It refers to making comparisons to the active self-presentation from other online users in terms of abilities, achievements, appearance, etc. (Feinstein et al., 2013; Vogel et al., 2014). In social networks, individuals pay more attention to their personal image on social platforms and consciously present themselves by publishing text, pictures, videos, and other information (impression management). On the one hand, most of the photos and other information that people display on social networks are carefully selected and edited to present their positive and beautiful sides (Fox and Vendemia, 2016) and to highlight their positive and idealized self-image (Krämer and Winter, 2008). This kind of self-presentation is controlled and strategic (Deri et al., 2017) and presents an illusion to the audience who browses the information: other people’s lives are better and happier (Schmuck et al., 2019). Previous research has found that upward social comparison is not only related to positive behaviors such as learning and improving one’s self (Suls et al., 2002), but also has a negative impact on individuals’ feelings of self-worth and their behavior (Lee et al., 2020) under certain circumstances. Geng et al. (2021) directly explored the relationship between social comparison tendencies and cyberbullying, and their study found that individuals with high levels of social comparison were more likely to feel envious on SNS, and further tended to bully others and be bullied online when they were dissatisfied with their bodies. Based on the content of their study we can assume that it is the upward social comparison in social comparison that has an effect on cyberbullying, but no further distinction is made between social comparison in their study. Therefore, in this study, we further investigate the effect of upward social comparison on cyberbullying (Patchin and Hinduja, 2011; Vogel et al., 2014).

In addition, according to General Strain Theory (GST), stressful events in life cause people to produce negative emotions, such as anxiety and depression, and individuals may engage in deviant behaviors to relieve emotional stress (Agnew, 1992). Social comparison on social media networks has an important impact on individual emotions (Choi et al., 2014). Upward social comparison on the Internet may induce negative emotions and create frustration in individuals. According to the contrast effect of upward social comparison, when individuals feel that they cannot reach the level of the social comparison object in the future, their self-evaluation level moves away from the social comparison goal (Rheu et al., 2021). Individuals will lower their self-evaluation level when facing upward comparison information (Blanton, 2001) and further induce individual depression (Chou and Edge, 2012). Poynor (2010) believes that when individuals compare themselves with others, they will have feelings of guilt (when they are higher than the other person) and jealousy (when they are lower than the other person) and may trigger corresponding aggressive behaviors (Livazović and Ham, 2019). When individuals perform cyber-upward social comparisons, negative emotions will be generated. To vent their negative emotions and achieve self-regulation, they might tend to implement cyberbullying behaviors (Barsky, 2011). Based on this, this research proposes the following hypothesis:

*Hypothesis 1*: There is a positive correlation between cyber upward social comparison and cyberbullying behavior.

### The mediating role of moral justification

“Moral justification” refers to individuals’ endowing their immoral behaviors with social value and moral purpose to obtain legitimacy for their bullying behavior and sometimes with a noble psychological evasion method (Barsky, 2011). Moral justification is one of the eight mechanisms of moral disengagement, similar to the concept of “neutralization,” which embodies the individual’s neglect and cover-up of unethical behaviors. It is a common psychological mechanism for individuals to implement unethical behaviors and corrupt behaviors (Yang et al., 2010a). The cyber upward social comparison makes people focus on their own shortcomings and produces lower self-assessment, which leads to more negative emotions and more unethical behaviors (Li, 2018; Suárez Vázquez et al., 2021). A large number of studies have also confirmed this notion (Walker et al., 2017). Upward social comparison will not only induce individual jealousy and lead to depression (Li, 2018) but also negatively predict the individual’s self-value (Vogel et al., 2014) and produce unreasonably low cognition of oneself (Fabian et al., 2018), which further produces the motivation for moral justification (Fang et al., 2022).

Moral disengagement is one of the most studied variables that affect cyberbullying. According to the theory of moral disengagement (Bandura et al., 1996), most people have established a personal code of moral behavior to regulate and guide their own behavior and suppress unethical behavior. As long as people’s behavior violates these guidelines, guilt and shame will be generated to prevent unethical behavior. However, moral disengagement helps those who violate their own moral code to reduce this feeling. This result is mainly achieved by changing people’s perceptions, that is, moral justification. Specifically, cyberbullying can be cognitively reconstructed through moral justification so that it appears to be less harmful or completely harmless to others (Yang et al., 2021). Individuals with moral justification endow their immoral behaviors with social value and moral purpose to obtain legitimacy for their bullying behavior and sometimes with a noble psychological evasion method (Zych, 2018). A large number of studies have proven the relationship between moral disengagement and cyberbullying (Lo Cricchio et al., 2021). Individuals with high levels of moral disengagement are more likely to participate in cyberbullying (Wang X. et al., 2016). As the most important dimension of moral disengagement, moral justification in the online environment may have an important role in promoting cyberbullying through belittling the bullied and beautifying bullying behavior. Therefore, this research proposes the following hypothesis:

*Hypothesis 2*: There is a positive correlation between cyber upward social comparison and individual moral justification; there is a positive correlation between moral justification and individual cyberbullying behavior; moral justification plays a mediating role between cyber social comparison and cyberbullying.

### The moderating role of social support

Online social support refers to the process by which individuals are respected, supported, and understood in online interpersonal interactions (Kessler et al., 1985; Turner et al., 2001) People with good online social support receive care, love, and support from group members in the virtual space and has a supportive environment that can have a positive impact on their self-esteem, which is the opposite with bulling behavior (Amanhuri and Lestari, 2021). There are a number of reasons why online social support might moderate the interrelations among upward social comparison, moral justification, and cyberbullying. First, greater online social support may help alleviate the negative psychological impact of upward social comparison, potentially reducing the likelihood that an individual engages in cyberbullying to regulate negative emotions. Cohen and Wills (1985) proposed the buffering effect model of social support to explain the mechanism of social support. They believe that social support can buffer the negative impact of potentially stressful events on individual physical and mental conditions. In the stress interaction model, Lazarus and Folkman (1987) suggest that when facing negative life events, problem-solving-oriented coping strategies (such as seeking social support) can effectively reduce the negative impact of the event on individuals (Li, 2017). When individuals have negative emotions and behaviors due to cyber upward social comparisons, online social support can play a buffering role in this procedure.

Second, online social support may help reduce the tendency to engage in moral justification on social media. When individuals receive online social support, they deal with the negative effects of cyber upward social comparisons more effectively, thereby affecting moral justification. Studies have found that young people will actively seek social support when facing negative online events (Pabian, 2019). Sufficient online social support may provide solutions to difficulties and emotional comfort to them. In other ways, it reduces the individual’s stress response or directly affects the physiological process, thereby reducing the adverse effects of stress physically and mentally. When cyber upward social comparison has a negative impact, online social support can buffer the pressure brought by cyber upward social comparison, maintain the individual’s mental health, and then be able to appropriately resolve the extremes of moral justification and help individuals make correct judgments, consequently reducing the level of moral justification.

Additionally, online social support has been positively correlated with altruistic orientation. High level of online social support can help individuals reduce moral justification in social networking platforms, thereby diminishing offensive behaviors and participation in cyberbullying behaviors (Aryn and Enrico, 2020). When individuals have a higher level of moral justification, they feel the support and recognition from the environment, and they are more aggressive and tend to accuse others, leading to more cyberbullying behaviors (Thornberg and Jungert, 2014). However, when individuals receive a high level of online social support, they feel the support and recognition from the environment, which can effectively improve their altruistic orientation, and they will reduce the occurrence of cyberbullying. Research by Li (2017) also shows that a high level of peer support and acceptance can effectively buffer individuals’ moral disengagement behaviors, thereby further reducing their aggressive behavior.

In other words, the prediction of cyber upward social comparison on cyberbullying is moderated by the support of the online society. Individuals with higher online social support will reduce cyberbullying behavior by decreasing the negative emotions brought about by cyber upward social comparisons and reducing moral justification. In view of this, this research proposes the following hypothesis:

*Hypothesis 3*: Online social support can effectively moderate the relationship between cyber upward social comparison and moral justification.

### The current study

**Figure 1:**
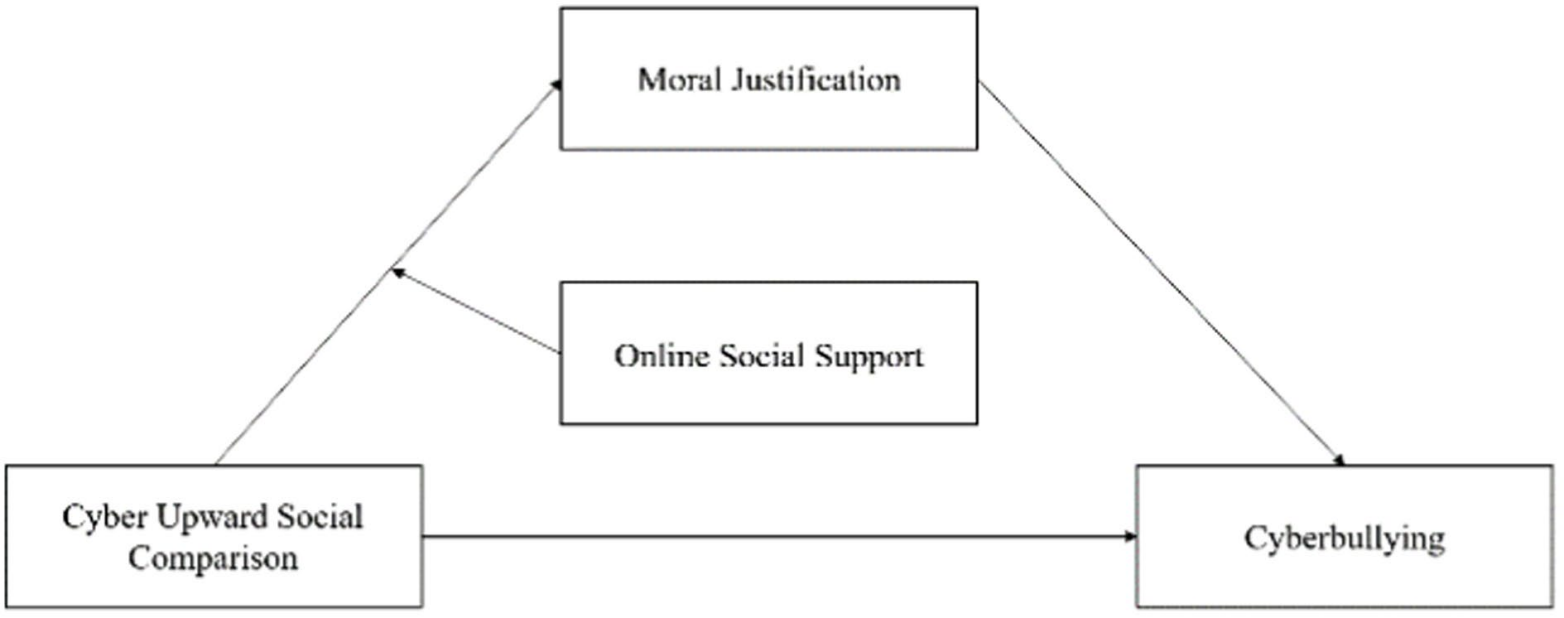
The model of the current study.

In summary, based on the GST and the buffering effect of social support, this research constructs a moderated mediation model. It verifies the relationship between cyber upward social comparisons and college students’ cyberbullying behavior for the first time and explores how moral justification predicts this procedure. The mediating role of online social support is evaluated. In other words, the effect of cyber upward social comparison on college students’ cyberbullying behavior can be described by a moderated mediation model. The hypothetical model is shown in Figure 1. In addition, the results of this study will provide references for the prevention and intervention of cyberbullying.

## Materials and methods

### Participants

In May 2021, we first communicate with two teachers from two Chinese university, and get their agreement and support. All participants are in good mental health, have no history of drug use and mental illness, have normal intelligence, right-handed, and individuals with mental illnesses from their immediate family members are excluded. All the participants completed the questions. A total of 294 students who did not pass the attention checks or failed to provide a valid age and social media use time were excluded from the study, and their data were not included in the final analysis. Finally, the questionnaire responses of 660 participants in China were retained. Participants ranged in age from 17 to 21 years old (*M* = 18.88 years, *SD* = 0.75). Taking into account the impact of time spent on social media on cyberbullying, we also measured the average amount of time participants spent on social media sites per day during the previous week (*M* = 157.44 min, *SD* = 145.85). In statistical power calculation, we follow the opinion of Hou et al. They believe the number of samples should be 5 fold the number of items. This study includes 51 items, and the required sample size was 255. Thus, the sample selection was in line with the requirements of this study. The distribution of demographic variables of the sample is shown in Table 1. All these demographic data were collected to comprehensively describe the research model to reveal its relationship with cyberbullying. The study protocol was approved by the Ethics Committee of Shandong Normal University.

**Table 1:** Demographic variables.

Demographic variables	Categories	Percent (%)
sex	Male	51.5
	Female	48.5

social media use time	<100 min	41.22
	100 min-1,000 min	58.78
Singleton	Only child	40.8
	Not an only child	59.2

family monthly income	Under 1,000 yuan	2.3
	1,000–2000 yuan	3.9
	2000–3,000 yuan	7.8
	3,000–4,000 yuan	12.0
	4,000–5,000 yuan	10.6
	5,000–6,000 yuan	13.4
	6,000–7,000 yuan	9.8
	7,000–8,000 yuan	8.1
	8,000–9,000 yuan	8.1
	9,000–10,000 yuan	6.1
	Above 10,000 yuan	17.9

Family origin	Countryside	50.6
	Township	8.3
	County town	17.8
	Prefecture level urban area	19.8
	Provincial capital cities and above	3.6

### Measures

#### Cyber upward social comparison

We used the upward comparison subscale of the Iowa - Netherlands Comparison Orientation Measure, which was compiled by Gibbons and Buunk and revised by Lian et al. (Gibbons and Buunk, 1999; Lian et al., 2017). The questionnaire contains 6 questions, which must be compared in the context of social networking sites (QQ space, WeChat Moments or microblog, etc.). A typical example is “on social networking sites, I often like to compare with those who live better than myself.” We used a 5-point scale, with 1 meaning “strongly disagree “and 5 meaning “strongly agree “. The higher the score on the scale, the more individuals tend to make social comparisons on social networking sites. Cronbach’s α was 0.96 in this study. The CFA displayed an excellent fit, χ2(47) = 115.45, *p* < 0.001, CFI = 0.99, TLI = 0.97, and SRMR = 0.03.

#### Moral justification

We used the moral justification scale in the adolescent moral disengagement scale compiled by Bandura et al. (1996) and revised by Yang et al. (2010b). The questionnaire contains four questions. A typical example is “you can beat those guys who abuse you.” A 5-point scale was adopted, with 1 meaning “strongly disagree” and 5 meaning “strongly agree.” The higher the score, the higher the level of moral justification. Cronbach’s α was 0.76 in this study. The CFA displayed an excellent fit, χ2(51) = 330.45, p < 0.001, CFI = 0.92, TLI = 0.90, and SRMR = 0.06.

#### Cyberbullying

The cyberbullying scale compiled by Erdur-Baker and Kavsut (2007) and revised by Zhou et al. (2013) was adopted. The cyberbullying scale measures the level of bullying in the online environment, such as “spreading someone’s rumors on the Internet.” The scale contains 18 questions and adopts a 4-point scale, ranging from 1 “never” to 4 “more than five times.” The higher the score, the higher the level of individual cyberbullying behaviors. Cronbach’s α was 0.78 in this study. The CFA displayed an excellent fit, χ2(9) = 346.84, p < 0.001, CFI = 0.93, TLI = 0.89, and SRMR = 0.03.

#### Online social support

We used the online social support questionnaire compiled by Liang and Wei (2008). A typical example is “when I feel lonely, I can talk to others through the Internet.” The questionnaire contains 23 questions, including four dimensions: friend support, emotional support, instrumental support, and information support. Likert’s 4-point scoring was adopted. The higher the score, the higher the individual’s level of online social support. Cronbach’s α was 0.94 in this study. The CFA displayed an excellent fit, χ2(486) = 1219.46, *p* < 0.001, CFI = 0.93, TLI = 0.91, and SRMR = 0.05.

### Procedure

We used cross–sectional design and recruited participants in class QQ group from two Chinese universities, and they answered the questionnaire through www.wjx.cn. Prospective participants were required to own and have used a mobile phone or computer to connect to the internet in the previous week. Data collection began in May 2021 and ended in June 2021, lasting a total of one month.

Before this test, all participants were informed about the purpose, procedures and measurements, potential risks, and possible benefits of the study before the survey. They were also informed that all the participants’ confidentiality would be protected in this anonymous survey research. Electronic informed consent was required from all the respondents for participation in the study. Participants could withdraw from the study at any time but were required to answer all the questions if they chose to finish the research.

Then, they were asked to complete the demographic information, including sex, age, singleton, family monthly income, family origin, and social media use time (the detail see Table 1). Finally, the participants completed the questionnaires, and we thanked for participants.

### Data processing

We used SPSS 19.0 software to analyze the data and Mplus 8.0 (Muthén & Muthén, Los Angeles, CA, USA) to Structural Equation Modeling analyze and verify the model, and SPSS’s PROCESS macro to perform the further simple slope analysis. In addition, this study also controlled for some variables, such as sex, age, singleton, family monthly income, family origin, and everyday social media use time.

### Common method biases test

Data collected using self-report may lead to common methodological bias. The study was controlled by using an anonymous survey and reverse scoring of some questions. At the same time, the Harman single-factor test was used to test the deviation of the common method. The results show that there were 12 factors with characteristic roots greater than 1, and the cumulative variation explained by the first factor was only 19.09%, which is less than the critical value of 40%. This shows that there was no serious common method deviation in this study (Zhou and Long, 2004).

We used Mplus 8.0 (Beijing Tianyan Rongzhi Software Co., Ltd., Beijing, China) to conduct CFA. The hypothesized four-factor model *χ^2^*(1646) = 1944.67, *p* < 0.001, root-mean-square error of approximation (RMSEA) = 0.02, comparative fit index (CFI) = 0.98, Tucker-Lewis index (TLI) = 0.98, and standardized root-mean-square residual (SRMR) = 0.04, displayed an excellent fit to the data. We further examined several alternative measurement models and compared them with the four-factor model. As shown in Table 2, the four-factor model fits our data better than other models, suggesting that our respondents could clearly distinguish the focal constructs.

**Table 2:** Results of confirmatory factor analysis of the measurement models.

Model	*χ* ^2^	df	*χ*^2^/df	RMSEA	CFI	TLI	SRME
Four-factor (A,B,C,D)	1,386.63	941	1.47	0.03	0.98	0.98	0.04
Three-factor (A + B, C, D)	1,2234.22	1,221	10.02	0.11	0.61	0.60	0.09
Two-factor (A + B + C, D)	16,437.45	1,223	13.44	0.13	0.47	0.44	0.14
One-factor (A + B + C + D)	20,945.34	1,224	17.11	0.14	0.31	0.28	0.15

A means moral justification; B means cyberbullying; C means online social support; D means cyber upward social comparison.

## Results

### Correlation analysis of demographic variables, cyber upward social comparison, moral justification, cyberbullying, and online social support

The correlation analysis results in this study showed that there is a significant positive correlation between cyber upward social comparison and moral justification, cyberbullying, and online social support, which indicated that cyber upward social comparison moves in the same direction as moral justification, cyberbullying, and online social support. Moral justification has a significant positive correlation with cyberbullying. To improve the accuracy of research results, the demographic variables, such as sex, age, family monthly income, singleton, family origin and social media use time were strictly controlled in the process of Mplus analysis (Table 3).

**Table 3:** Descriptive statistics of each variable and correlation matrix.

Variables	*M*	SD	1	2	3	4	5	6	7	8	9	
Sex	1.48	0.50	1									
Age	18.88	0.75	−0.06	1								
Singleton	1.62	0.49	0.23**	0.07*	1							
Family monthly income	6.70	2.81	−0.19**	−0.17**	−0.27**	1						
Family origin	2.16	1.33	−0.06	−0.10**	−0.46**	0.43**	1					
Social media use time	157.44	145.85	0.12**	0.01	0.09**	0.00	0.00	1				
Cyber upward social comparison	19.07	4.52	−0.02	−0.07*	0.03	0.07	0.01	0.06	1			
Moral justification	1.87	0.67	−0.15**	−0.12**	−0.01	0.02	−0.12*	0.04	0.23***	1		
Cyberbullying	18.85	2.02	−0.10**	−0.02	0.05	0.05	−0.00	0.12**	0.27**	0.33***	1	
Online social support	3.56	0.63	−0.05	0.24**	−0.18**	0.12**	0.24**	−0.01	0.07	−0.20***	−0.05	1

^**^p < 0.01; ^***^p < 0.001; M, Mean; SD, Standard Deviation.

### Test for structural model

**Figure 2:**
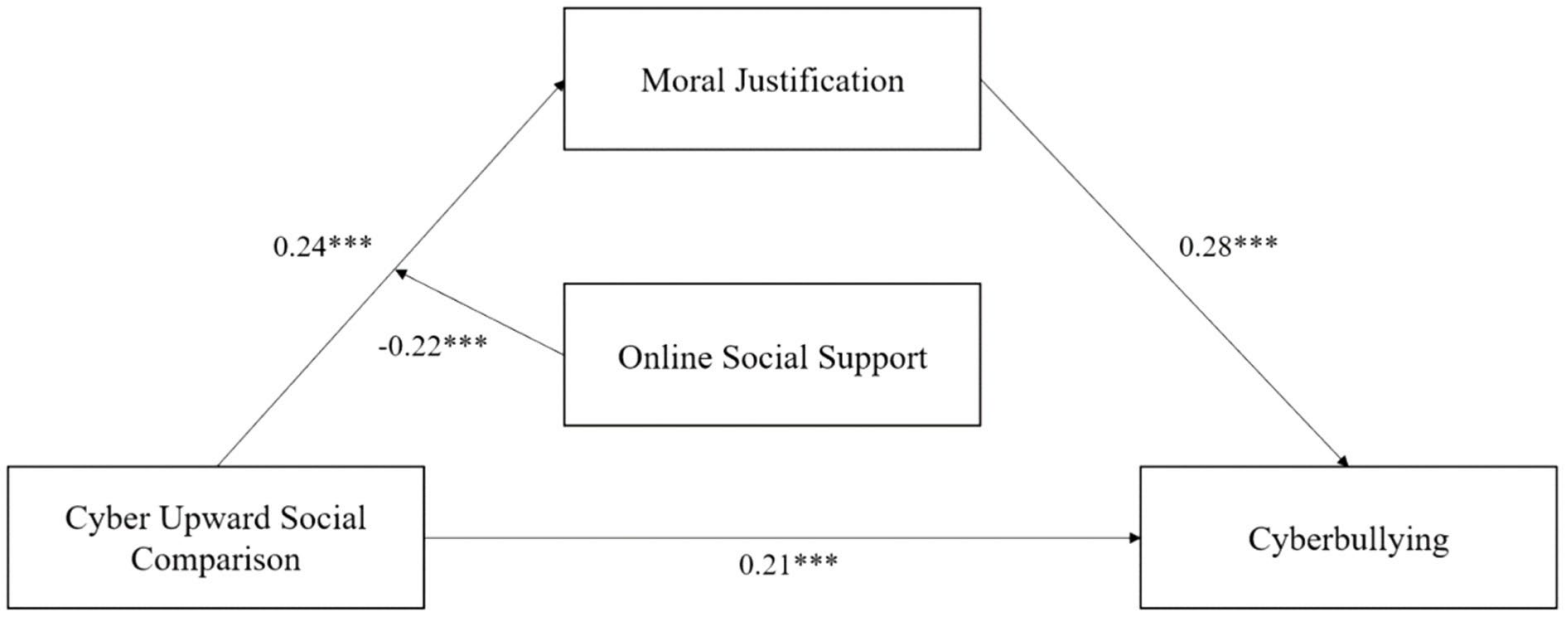
The model of the current study with metrics.

The structural equation model was used to investigate the mediating effect of moral justification and moderating effect of online social support. The maximum likelihood estimation method is used to estimate the moderated mediation effect. As shown in Figure 2, cyber upward social comparison (*β* = 0.24, *p* < 0.001) and moral justification (*β* = 0.28, *p* < 0.001) positively predicted cyberbullying. After putting online social support into the model, the interaction between cyber upward social comparison and online social support has a significant predictive effect on moral justification (*β* = −0.22, *p* < 0.001), which shows that online social support can play a moderating role in the prediction of moral justification by cyber upward social comparison, which was shown in Figure 2. The results of bootstrap revealed that cyber upward social comparison had significant direct effect on cyberbullying (direct effect = 0.21, SE = 0.03, *p* < 0.001, 95% bootstrap CI [0.165, 0.252]), and significant indirect effects on cyberbullying *via* moral justification (indirect effect = 0.07, SE =0.01, *p* < 0.001, 95% bootstrap CI [0.048, 0.092]). These results indicated that self-compassion and depression mediated the relation between overparenting and NSSI behaviors, which were shown in Figure 2.

**Figure 3:**
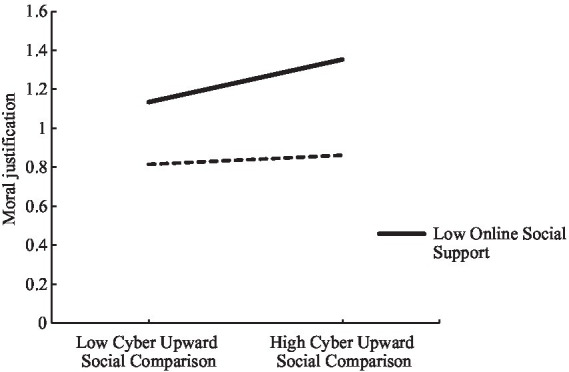
The moderating effect of online social support on the relationship between cyber upward social comparison and moral justification.

Further simple slope analysis shows that for participants with high social support (*M + 1*SD), cyber upward social comparison cannot predict moral justification, simple slope = 0.0073, *t* = 1.3372, *p* = 0.1815; for participants with low social support (*M − 1SD*), cyber upward social comparison has a significant positive predictive effect on moral justification, simple slope = 0.0287, *t* = 4.9625, *p* < 0.001. It shows that with the improvement of online social support, the predictive effect of cyber upward social comparison on moral justification gradually decreases, and the mediating effect of moral justification on the relationship between cyber upward social comparison and cyberbullying also shows a downward trend (Figure 3).

## Discussion

In this study, we built a moderated mediation model to explore the relationship between cyber upward social comparison and cyberbullying and further explored the mediating role of moral justification and the moderating role of online social support. The results show that there is a significant correlation between cyber upward social comparison and cyberbullying. Cyber upward social comparison predicts cyberbullying through moral justification, and this indirect path is moderated by online social support. The findings of this study help to answer the two key questions of how and when (under what conditions) cyber upward social comparison predicts cyberbullying behavior, which have certain theoretical and practical significance.

### The relationship between cyber upward social comparison and cyberbullying

This study found that there is a significant positive correlation between cyber upward social comparison and cyberbullying, which is consistent with Hypothesis 1. The relationship between cyber upward social comparison and cyberbullying has also been reflected in previous studies, which have demonstrated that cyber upward social comparison not only has a negative impact on individual psychological health (Erdur-Baker and Kavsut, 2007; Yang et al., 2010b; Aboujaoude et al., 2015), such as damaging people’s mental health (Zhou et al., 2013), reducing happiness (Zheng et al., 2020), and producing negative emotions of jealousy and depression (Liang and Wei, 2008; Lee et al., 2020), but can also lead to irrational cognition and behavior (Aliyev and Gengec, 2019). A study of adolescents found that social comparison was associated with severe bullying behavior (Stasio et al., 2016). The researchers found that social comparison among adolescents led to peer competition and, in the process of competition, to the maximization of their own gains relative to others and, thus, to intentional harm behavior. This leads to the occurrence of individual bullying behavior (Boer et al., 2021). Schlosser and Levy also found through experimental research that upward social comparison can reduce people’s helping behavior (Schlosser and Levy, 2016). These studies all support the relationship between cyber upward social comparison and bullying behavior in different aspects.

In addition, the results of this study also support the general strain theory model. Negative emotions, such as depression, caused by individuals in the face of cyber upward comparison often cause individuals to engage in deviant (aggressive) behaviors to relieve the pressure brought on by negative emotions (Agnew, 1992; Patchin and Hinduja, 2011; Zhang et al., 2018). Faced with the pressure of upward social comparison on the Internet, individuals may be more aggressive, which manifests as cyberbullying in the online environment. From the available literature retrieved, this study is the first to explore the relationship between cyber upward social comparison and cyberbullying, which provides an important supplement to the literature on the influencing factors of cyberbullying and further improves the relevant research on cyberbullying.

### The mediating role of moral justification

This study also found a mediating effect of moral justification, that is, cyber upward social comparison may lead to individual moral justification, which further leads to individual cyberbullying behavior, which is consistent with Hypothesis2. This finding helps to explain the mechanism by which cyber upward social comparison predicts cyberbullying behavior.

On the one hand, consistent with the studies available, previous researchers have found that cyber upward social comparison will have an impact on people’s cognition (Verduyn et al., 2020), resulting in cognitive distortion. Moral justification is a kind of irrational cognition, which is the individual’s reinterpretation of the behavior that hurts others (Barsky, 2011). Therefore, it is easier for individuals to engage in moral justification when they make cyber upward social comparisons. As van de Ven (2017) found that people can experience negative cognitions and emotions such as malicious type of envy when someone else is better than them, which could produce a motivation to pull down or attack others and rationalize own behaviors. On the other hand, as one of the mechanisms of moral disengagement, moral justification can affect the pro-organizational non-ethical behavior of employees and is one of the most important predictors of cyberbullying (Yao et al., 2021). A large number of previous studies have shown that individuals who adopt moral disengagement are more likely to participate in cyberbullying (Wang et al., 2017). According to social cognitive theory, cyber upward social comparison can reconstruct the cognition of cyberbullying through moral justification so that cyberbullying behavior seems to be less or not harmful to escape the self-punishment caused by violating inner norms (Aboujaoude et al., 2015). In daily life, many people also think nothing of bullying on the Internet, believing that it is a virtual environment that has no impact on reality, thus increasing the possibility of participating in cyberbullying. This study explores the mediating mechanism of moral justification between cyber upward social comparison and cyberbullying, which is helpful to address the influential path of cyber upward social comparison on college students’ cyberbullying behavior.

### The moderating role of online social support

This study also found that the mediating role of moral justification between cyber upward social comparison and cyberbullying was moderated by online social support, which moderated the indirect path between cyber upward social comparison and moral justification and cyberbullying, which was also consistent with Hypothesis 3. This study is consistent with previous studies that have shown that social support has a significant impact on moral justification. Shen et al. (2019) explored the impact of social support on moral disengagement through emotion. They found that individuals with high social support elicited less anger and hostility and were less involved in moral disengagement, of which moral justification is one of the most important dimensions. In addition, studies have found that online social support has a positive impact on people’s mental health (Perry et al., 2018; Kamalpour et al., 2020). According to Buffering Model Theory, social support can alleviate the negative emotions generated by individuals suffering from adverse life events, so people with high social support will feel less stress and negative emotions and less moral justification (Pelton et al., 2004; Fida et al., 2015; Campaert et al., 2017).

According to previous research, social support is an important resource to protect individuals from bullying and its negative effects (De Beer, 2014; Rossiter and Sochos, 2018). Therefore, a high level of social support in social networking platforms environment plays an important role in reducing aggressive behaviors and increasing prosocial behaviors (Calvete et al., 2010). Individuals with higher levels of online support may experience lower stress and fewer negative emotions and be relieved of individual physiological and emotional reactions in the face of stressful events to improve unreasonable cognition, lower levels of moral justification, and decrease involvement in cyberbullying. This conclusion is of great significance for individuals to avoid cyberbullying and promote the development of psychological adaptation.

### Practical value

This study has implications for reducing cyberbullying behavior in the online environment. First, a correlation was found between cyber upward social comparison was found to be positively associated with and cyberbullying. In society, we should strengthen the supervision of online information release in the process of network management, reduce false online information content, such as bragging and comparing, and decrease cyber upward social comparison (Weinstein, 2017). We should build a harmonious and warm network environment by encouraging and promoting healthy and real network information and promote positive mental attitudes to make network participants aware of the negative effects of upward social comparison on the Internet and jointly promote the reduction of cyberbullying.

Second, the study found that cyber upward social comparison can predict cyberbullying through moral justification, which indicates that cyber upward social comparison can make people feel comfortable participating in cyberbullying by distorting the cognition of cyberbullying events and making seemingly moral justification for their own cyberbullying behaviors (Barsky, 2011; Verduyn et al., 2020). Individuals with high levels of moral justification are more likely to rationalize their aggressive behavior, thereby increasing cyberbullying. For example, many people argue that social networking platform is virtual and will not cause harm to reality. This suggests that we should strengthen online education for internet users, make internet users realize the real harm of cyberbullying, and help them understand the real trouble caused to the victims to reduce the occurrence of cyberbullying. And through corresponding targeted measures to improve the level of individual moral sense, reduce moral justification. For example, victims of cyberbullying can be interviewed to describe the harm caused by cyberbullying to them and the impact of cyberbullying on their real life and other measures to reduce moral justification.

Finally, we found that online social support moderated the mediating role of moral justification between cyber upward social comparison and cyberbullying. Online social support provides individuals with timely and sufficient psychological satisfaction, and makes up for the relative deprivation caused by upward social comparisons, thereby effectively reducing cyberbullying. In other words, individuals with good online social support were less likely to use moral justification and less likely to participate in cyberbullying. In daily life, people communicate more with others through the Internet (Kamalpour et al., 2020), which inspires us to build harmonious interpersonal relationships in the internet environment and to communicate more with others through the Internet, rather than making meaningless comparisons. At the same time, building an online social support network can effectively improve the level of individuals obtaining social support. Future research can try to establish a corresponding online social support-related website, which can help individuals more easily obtain social support.

### Limitations and future prospects

The limitations of this study are as follows. First, from the perspective of the research sample, this study only focuses on college students, without further exploration in other groups. Although previous studies have proven that college students are representative to a certain extent, future studies can expand the sample and further explore the impact of cyber upward social comparison on cyberbullying by targeting groups of different ages and occupations.

Second, from the perspective of research content, on the one hand, this study only considered the effect of cyber upward social comparison on cyberbullying, but individual personal characteristics such as self-esteem, empathy are not considered. So in the future studies we should further consider the individual characteristic factors and environmental factors into the integration model of joint consideration, constructing an integrated model of cyberbullying. On the other hand, this explores the mediating effect of moral justification on cyber upward social comparison and cyberbullying. Moral justification is an important dimension of moral disengagement. Other dimensions of moral disengagement refer to the disengagement methods used by individuals to eliminate their own responsibilities. Given, it is necessary for future research to further examine the role of other dimensions of moral disengagement in cyber upward social comparison and cyberbullying.

In addition, in terms of research methods, participants engaged in self-report, which may have resulted in a social approval effect. Given the disadvantages of self-reporting, the accuracy of the study data may be affected. Although this study was controlled by setting attention checks questions and adjusting the sequence of questions, future studies need to adopt multisubject reporting and variable measurement at different periods to enhance the accuracy and reliability of the data.

Finally, although this study established a moderating mediating model of the prediction of upward social comparison on college students’ cyberbullying behavior, it was a cross-sectional study and could not show a causal relationship between variables. Future research could further adopt longitudinal research to investigate the exact relationship between variables and further clarify the intervention entry point.

## Conclusion

Using well-constructed questionnaires allows measure upward social comparison, moral justification, online social support and cyberbullying behaviors, based on GST, it was found that a moderated mediating model could be established, indicating the relationship between cyber upward social comparison and cyberbullying, the mediating role of moral justification and the moderating role of online social support. The following conclusions are drawn: (1) There is a significant positive correlation between cyber upward social comparison and cyberbullying; (2) Moral justification plays a mediating role in the relationship between cyber upward social comparison and cyberbullying; (3) The mediating effect of moral justification on the relationship between cyber upward social comparison and cyberbullying was moderated by online social support. Under the condition of high online social support.

Our finding suggested that focusing on upward social comparison could be a new and valuable direction in developing cyberbullying-related prevention and intervention programs. Cyberbullying is commonly a type of covert behaviors; targeting social comparison and moral justification is important for moving forward research to improve cyberbullying behaviors.

## Data availability statement

The raw data supporting the conclusions of this article will be made available by the authors, without undue reservation.

## Ethics statement

The studies involving human participants were reviewed and approved by the Ethics Committee of Shandong Normal University. The patients/participants provided their written informed consent to participate in this study.

## Author contributions

YF, HW, and XK contributed to conception and design of the study, wrote the first draft and sections of the manuscript. HW and XK organized the database and performed the statistical analysis. All authors contributed to the article and approved the submitted version.

## Conflict of interest

The authors declare that the research was conducted in the absence of any commercial or financial relationships that could be construed as a potential conflict of interest.

## Publisher’s note

All claims expressed in this article are solely those of the authors and do not necessarily represent those of their affiliated organizations, or those of the publisher, the editors and the reviewers. Any product that may be evaluated in this article, or claim that may be made by its manufacturer, is not guaranteed or endorsed by the publisher.
